# RCC Immune Microenvironment Subsequent to Targeted Therapy: A Friend or a Foe?

**DOI:** 10.3389/fonc.2020.573690

**Published:** 2020-09-30

**Authors:** Wenjin Chen, Xiuwu Pan, Xingang Cui

**Affiliations:** Department of Urology, The Third Affiliated Hospital of Second Military Medical University, Shanghai, China

**Keywords:** renal cell carcinoma, tumor microenvironment, immune cells, targeted therapy, immunotherapy

## Abstract

Renal cell carcinoma (RCC) is composed of different subtypes with distinct molecular and histological tumor heterogeneity. Although the advent of various targeted therapies has improved the survival of patients with advanced RCC over the past 15 years (since 2006), few cases experienced complete response due to drug resistance. Recent studies have demonstrated that the outcomes following targeted therapies are potentially associated with intricate cross-links between immune responses and suppressors in the tumor microenvironment (TME). In addition, progress on drug research and development enhances our awareness and understanding about immunotherapy and combined treatment. In this review article, we intend to make a comprehensive summary about TME and its alterations following targeted therapies, provide valid evidence in this aspect, and discuss optimal matches between targeted therapy and immunotherapy.

## Introduction

Kidney cancer is one of the leading cancer types in the United States, with estimated 73,750 new cases and 9,860 deaths by both sexes in 2020, accounting for 4.1% of all new malignancies (1). Renal cell carcinoma (RCC) accounts for about 85% of kidney cancer (2). The currently approved medicaments for targeted therapy comprise vascular endothelial growth factor (VEGF) inhibitors such as bevacizumab; tyrosine kinase inhibitors (TKIs) such as sunitinib, pazopanib, sorafenib, cabozantinib, and axitinib; and mammalian target of rapamycin (mTOR) inhibitors such as temsirolimus and everolimus (2–4). However, the overall outcomes remain unsatisfied despite the remarkable progress of the current targeted therapies in increasing the 5-year survival rate, especially for high-risk patients with respect to the clinical grades such as the aggressive clear-cell RCC (ccRCC) (5, 6).

Among the pan-carcinomas, RCC ranks one of the tumors with the highest degree of immune infiltration (7). Therefore, this category of solid tumors has been generally recognized as insensitive to chemotherapy and is expected to be responsive to immunotherapy, which may indicate that the era of cytokine-based therapy is around the corner, although the reported responsive rate is only about 10% (8, 9). Immune checkpoint inhibitors (ICIs) have made great progress and shown definite value in patients with RCC through the years, whether or not patients had received treatment previously (10, 11). Some recent large randomized controlled trials (RCTs) have validated that ICIs combined with TKI-targeted therapy are superior to the conventional VEGF-targeted agent sunitinib (12, 13). Therefore, it seems to be more accepted that targeted therapies potentially help the immune response through regulating abnormal tumor vascularization (14). However, antiangiogenic therapies may lead to reprogramming of the tumor microenvironment (TME) while resisting vascularization associated with tumor progression (15). On the other hand, these RCTs compared pairing axitinib and ICIs with sunitinib, whose results could partly originate from the superior activity of axitinib vs. sunitinib. Meanwhile, pazopanib, and sunitinib combined with ICIs have been demonstrated to bring about more adverse events (13, 16). Thus, it is worthwhile to investigate whether the diversities in safety and therapeutic efficacy are due to changes in TME following targeted therapy.

In this review article, we would provide current evidence and emerging concepts to demonstrate that the angiogenesis-directed therapies interact with the immune TME and affect immune response in RCC and discuss some potentially promising combined treatment regimens that have been demonstrated to minimize the toxic effects and augment the benefits.

## Different Immune Cells in the Tumor Microenvironment

It has long been acknowledged that targeted therapy or even immunotherapy may be influenced by immune invasion in the TME (17). There are distinct types of immune cells involved with the TME and constituting the immune invasion mediated by tumor and immune regulation, including T cells, regulatory T cells (Tregs), tumor-associated macrophages (TAMs), myeloid-derived suppressor cells (MDSCs), and cancer-associated fibroblasts (CAFs) (18, 19).

### T Cells

In the immune atlas of RCC, there are 11 CD8+ phenotypes and eight subtypes of CD4+ T cells (20). The infiltration of both CD4+ and CD8+ T cells was found to be increased after anti-angiogenic treatment in RCC (21). An immunohistochemical study with 135 RCC patients (22) reported that CD8+ T cell infiltration and immune checkpoints could prospectively predict the prognosis of the disease. Another study (23) analyzed CD8+ T cells in 87 clinical cases and demonstrated that the density of CD8+ T cells with Tim-3 and PD-1 expression was correlated with RCC aggressiveness and grades. Meanwhile, Tregs (CD25+ cells) are the subset of CD4+ T cells and may become one promising target for immunotherapy. CD25 inhibitors could remove the Treg infiltration and coordinate with ICIs *in vivo* and *in vitro*, even in RCC patients (24). Tregs could regulate the tumor development and immune escape through interacting with the TME by inhibiting the maturation of antigen-presenting cells (APCs) and facilitating angiogenesis indirectly (25–27). Likewise, tumor proliferation and metastasis are also correlated with Treg mediation (28). Thus, the presence of T cells in TME plays both an antitumor role and a protumor role in tumor development. Currently available findings indicate that targeted therapy can regulate the T cell-related TME both positively and negatively (21). However, in order to explore appropriate treatments, we should further focus on illustrating the roles of various T cells in the RCC TME.

### Myeloid-Derived Suppressor Cells

MDSCs are derived from myeloid progenitor cells but lose the opportunity of differentiating into monocytes/macrophages or granulocytes (29), while they become monocytic MDSCs (M-MDSCs) and polymorphonuclear MDSCs (PMN-MDSCs) (30). MDSCs are described as a cell group dominating in tumor immune escape through suppressing the function of natural killer (NK) cells and T cells and promoting immunosuppressive Tregs (31, 32).

MDSC accumulation in RCC was found to be correlated with the expression of C-C motif ligand 2 (CCL2), interleukin (IL)-17, and IL-18 (33) and associated with the course of angiogenesis. Usually, myeloid cell differentiation would be blocked by tumor-associated products and VEGF, thus increasing the number of immature myeloid cells with heterogeneity (34). Then, MDSCs promoted angiogenesis and contributed to cancer cell viability and migration, enabling tumor metastasis (35). Additionally, MDSCs aggravated the suppressive immune TME by contacting with M2-TAMs through IL-10 and transforming growth factor (TGF)-β, which could be attenuated by downregulation of high-mobility group box-1 (HMGB1) in RCC progression (36). Therefore, eliminating MDSCs might reactivate the immune surveillance or killing function and decrease the immune tolerance of tumors.

### Tumor-Associated Macrophages

TAMs are derived from monocytes and play an important role in the TME as inflammation mediators. Heidegger et al. (18), Santoni et al. (37), and Kovaleva et al. (38) maintained that TAMs could promote neovascularization and facilitate tumor progression. In addition, they participated in tumor immune escape through secreting suppressive factors and induced tumor metastasis through extracellular matrix remodeling-related enzymes.

TAMs in RCC first caught the sight of researchers about 10 years ago because of the finding that high TAMs frequency and CD163 (one of the TAM surface markers) infiltration occurred in the cases with poorer prognosis data, when researchers cocultured RCC cell lines with polarized type II macrophages with CD163+ (39). Additionally, TAMs recruited monocytes to tumor compartments via CCL-2 and IL-10 but promoted immune escape (40). In addition, poor outcomes or easier relapses were found to be correlated with TAM frequency in clinical RCC cases (41). Higher inflammation of TAMs occurred in the TME of metastatic RCC as compared with the initial compartment (42). Likewise, the stimulation of angiogenesis by TAMs deserves discussion. A study comprising 51 RCC cases (43) reported that the number of microvessels was positively correlated with the frequency of TAMs. However, recent transcriptional profiling data of human TAMs supported that these heterogeneous macrophages in the TME were not limited to conventional M1 and M2 types, especially in ccRCC. This also reminds us that there are diversities in TAM characterizations among all types of RCC (44).

### Cancer-Associated Fibroblasts

Actually, CAFs are not immune cells substantially but closely interact with the immune TME and target agents through remodeling the primary structure of tumor compartments by producing cytokines. Thus, we will discuss this distinct type of cell population. CAFs are known for the chronic activation of primary fibroblasts via cancer-induced epigenetic pathways (45). Essentially, CAFs can promote angiogenesis and tumor growth by producing VEGF, TGF-β, fibroblast growth factor (FGF), platelet-derived growth factor (PDGF), and epidermal growth factor (EGF). In addition, immune cells produce IL-1β to stimulate the inflammation pathway in CAFs (46). An *in vitro* study (47) showed that the proliferation, viability, and migration of RCC cell lines were enhanced after coculture with CAFs. A retrospective study (48) reflected that the staining density of CAFs was positively correlated with tumor grades and poor prognosis. Importantly, CAFs were found to be correlated with ccRCC progression via the hypoxia-inducible factor 1-alpha (HIF-1α) (46), and the overexpression of HIF-1α has been demonstrated to be the driver factor of ccRCC (49). Similarly, the expression of FGF-2 could potentially predict the aggressiveness, which is exactly part of the CAF role (50). Additionally, CAFs could express fibroblast activation protein-a (FAP) in the TME (51), which was reported to be a biomarker of ccRCC in the previous study (48). Therefore, the role of CAFs in RCC TME deserves further investigation.

## Conversation Between Targeted Therapy and the Immune Tumor Microenvironment

**Figure 1:**
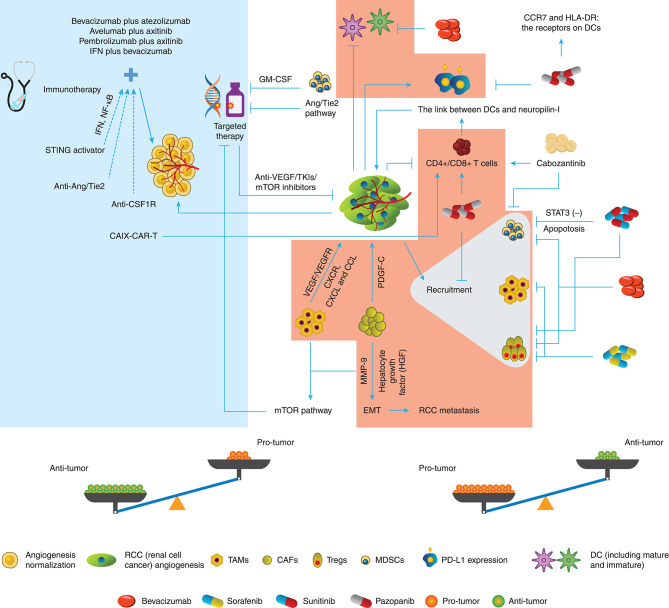
Conversation between targeted therapy and immune TME in RCC. The different immune cells in the TME of RCC promote or inhibit the angiogenesis through various pathways. Targeted therapy drugs can inhibit or promote the immune roles of immune cells besides block the angiogenesis. Therefore, the potential balance exists in the Anti-tumor role and Protumor role.

As has been discussed before, angiogenesis and related factors induce the reaction of immunity including immune response and immunosuppression, but on the other hand, immune TME helps angiogenesis sustenance. Hence, we tend to hypothesize that there is an interconnection between targeted therapy and immune TME, whose evidence was indeed ascertained (Figure 1).

### Alternations of the Immune Tumor Microenvironment Under Targeted Therapy

Firstly, dendritic cell (DC) immaturity occurs when VEGF binds to VEGF receptor (VEGFR)2, thus decreasing the antigen presentation and increasing the programmed death ligand 1 (PD-L1) expression in DCs. Subsequently, VEGF downregulates the T cell function by blocking CD4+ and CD8+ cell maturation. Moreover, tumor angiogenesis recruits more suppressive immune cells such as MDSCs, TAMs, and Tregs as stated above (52).

Fortunately, growing evidence has demonstrated that angiogenesis-directed treatment can reverse this suppressive effect (53), and the knockdown of VEGFR-1 can decrease the frequency of the suppressors in RCC (54). Likewise, Yamamoto et al. (55) and Guislain et al. (56) showed that sunitinib could reduce MDSC accumulation in the tumor compartment and improve the suppressive TME through blocking the signal transducer and activator of transcription 3 (STAT3) signaling pathways, thus increasing the apoptotic rate of MDSCs in mice. Coincidently, sunitinib could decrease the frequency of Tregs both in the TME and the whole body subsequently to the first cycle of treatment. In the next cycles, the Treg level decreased even further in patients with more Tregs before treatment (57). However, the effect of sunitinib on Tregs could be indirect because Tregs showed no further decline after 2-week sunitinib treatment (58).

On the other hand, sorafenib decreased the number of systemic and intratumoral Tregs and suppressed macrophages (59–61). Additionally, pazopanib reduced Tregs, monocytes, and MDSCs but stimulated T cell response (62). *In vitro*, less PD-L1 and more CC chemokine receptor 7 (CCR7) and human leukocyte antigen-DR isotype (HLA-DR) were detected in DCs from patients receiving pazopanib compared with the DCs from patients receiving sunitinib (63), indicating that an immunomodulatory effect of pazopanib could potentially improve responses and clinical outcomes of patients with mRCC, compared with sunitinib. In addition, cabozantinib enhanced the function of CD8+ and NK T cells but decreased MDSCs (64). Finally, studies on bevacizumab (65, 66) showed that this VEGF-targeted agent could stimulate the maturation of DCs and decrease Tregs in clinical trials and reduce MDSCs in the tumor site in animal models of RCC (67). Collectively, these studies have illustrated the multidimensional role of anti-VEGF-targeted therapy on the immune TME as possibly both facilitating and restraining sides of antitumor immunity.

### The Role of the Immune Tumor Microenvironment in Targeted Therapy

Natural and adaptive immune responses are well-regarded as the aspects to induce endothelial proliferation, which has become the double-edged sword for targeted therapy (68). Tumor-propelled inflammation produces proangiogenic cytokines to boost more inflammatory cells or factors to migrate to the tumor compartment. Besides the immunosuppressive cells such as TAMs stated above, neutrophils and DCs also can produce VEGF-related proangiogenic cytokines or VEGFR-related expression to weaken the antiangiogenic agents, including C-X-C chemokine receptor (CXCR), C-X-C motif ligand (CXCL), and CCL families (52). In addition, T cells facilitate neovascularization through intensifying the correction between DCs and neuropilin-I, instead of secreting VEGF directly (69).

Resistance to targeted therapy remains a crucial challenge in clinical cancer management. MDSCs from resistant murine tumors could confer resistance to anti-VEGF antibody therapy when they were added to sensitive tumors (70). The resistance of MDSCs to sunitinib is believed to be associated with granulocyte-macrophage colony-stimulating factor (GM-CSF) in RCC, but sunitinib combined with GM-CSF treatment still led to resistance to sunitinib both *in vivo* and *in vitro* (71). TAMs and CAFs were both demonstrated to participate in mTOR resistance (48), although little evidence has been sought in RCC presently. Moreover, CAFs can strengthen PDGF-C expression and shape resistance to targeted therapy, which surmounts the block of VEGF-directed angiogenesis (72). CAFs were found to promote tumor metastasis by elevating the expression of hepatocyte growth factor (HGF) and the accumulation of matrix metalloproteinase (MMP)-9 (73). High expression of peristromal hormone (PN) was detected in ccRCC, indicating the interlink between tumor cells and CAFs (74), while PN is believed to correlate with cell invasion and epithelial–mesenchymal transition (EMT) in RCC (75).

Immune TME modification under ICIs also influences the outcome of targeted therapy, especially in angiopoietin (Ang)/Tie2-directed angiogenesis. ICIs stimulate anti-Ang-2 activation, and patients with a high baseline level of that usually had poorer survival outcomes (76, 77). CD68+ and CD163+ macrophages are indicated that play a crucial role in Ang-2-mediated resistance, and ICIs could be the partner with Ang-2 inhibition. Moreover, bevacizumab combined with ipilimumab could reduce the Ang-2 expression (52, 78, 79). A clinical trial by Osama et al. is underway to investigate the effect of Ang-2 inhibitor and PD-1 inhibitor (NCT03239145), and their result about the correlation between the immune TME and angiogenesis-targeted therapy is anticipated. Although these data did not bring insights into the role of immune TME in targeted therapy directly, it is noted that both activated and inhibited pathways of angiogenesis can be influenced by aspects of the immune TME.

## Has the Age of Combination of Targeted Therapy and Immunotherapy Approached?

It is common sense that angiogenesis and immune tolerance matter a lot in the physical state until cancer induces them into pathological mechanisms. Considering the cross talk between immune regulation and angiogenetic modulation, combinations of IO and targeted therapy are currently expected to be the strategies to synergistically enhance the therapeutic efficacy. Although there are no comparative studies to explore different combinations in clinical trials, the clinically related diversities occurred in the preclinical studies (80–83). Thus, we need to be considerably cautious in selecting pairing agents based on mechanisms and preclinical trials.

### Current Combined Therapies of Targeted Therapy and IO

The first effort on ICIs plus targeted therapy can date back to NCT01984242, in which McDermott et al. (84) and Pal et al. (85) established the immune TME and angiogenesis profiling of 305 treatment-naive patients, and the responses of these patients to bevacizumab plus atezolizumab or sunitinib depended on the gene profile characteristics. Likewise, in a phase III trial performed by Rini et al. (86), the median progression-free survival (PFS) of the bevacizumab plus atezolizumab cohort was 11.2 months with 40% adverse events vs. 7.7 months in the sunitinib-alone cohort with 54% adverse events [hazard ratio (HR) 0.74].

A recently published study on advanced RCC involving 443 patients who received avelumab plus axitinib and 444 patients who were treated with sunitinib alone (NCT02684006) showed that avelumab plus axitinib significantly improved the PFS by 5–6 months regardless of the PD-L1 positive group or the overall population as compared with sunitinib alone. The objective response rate (ORR) of the combined treatment was also increased in the PD-L1-positive population (55.2 vs. 25.5%). However, both therapies led to adverse events in more than 99% adverse cases (13). In addition, the surprising result was also observed in the study of pembrolizumab plus axitinib vs. sunitinib alone. During a median 12.8-month follow-up period, pembrolizumab plus axitinib group showed a higher survival rate than sunitinib-alone group (89.9 vs. 78.3%, *P* < 0.0001). More importantly, pembrolizumab–axitinib combination benefits patients across the favorable, intermediate, and poor risk [International Metastatic RCC Database Consortium (IMDC) risk groups] instead of only PD-L1 expressive population (12).

Concerning interferon-alpha (IFN-α) plus targeted therapy, there is also a phase III study (NCT00631371) that investigated the efficacy of IFN plus bevacizumab vs. temsirolimus plus bevacizumab (87), finding no significant diversities existing in PFS, OS, and ORR, indicating that the combination of temsirolimus and bevacizumab was not superior to IFN plus bevacizumab in the first-line therapy for metastatic RCC. However, little is understood about the basic mechanisms of these synergic benefits from current combined therapies. Additionally, it is controversial that there are superiority and inferiority between combined therapies of completed clinical trials.

### Potential Optimal Match of Targeted Therapy and IO

Albeit the considerable medical value of anti-angiogenesis and ICIs, as they reprogram the TME process and craft such synergistic impacts, new targeted therapies and immunotherapy (IO) still require clinical assessment. We expect the advent of new concepts about targeted therapy and IO to guide the way of new agents that exert better outcomes in the coming years. Besides the clinical trials and the above results, several clinical studies on combinations are underway or on the way (Table 1).

**Table 1:** Current clinical trials targeting the IO combined with anti-angiogenesis therapy for RCC.

**NCT number**	**Interventions**	**Phase**	**Primary outcome measures**	**Status**
NCT04322955	Cabozantinib plus nivolumab	III	Complete response	Recruiting
NCT01218867	Anti-VEGFR CAR CD8+ PBL	I, II	Response rate	Terminated (no objective responses)
NCT00440973	Bevacizumab plus interleukin-2	II	Time to progression	Terminated (contract issues)
NCT03024437	Atezolizumab plus entinostat and bevacizuma	I, II	Dose of entinostat	Recruiting
			Objective response rate	
NCT02210117	Nivolumab, surgery	I	Incidence of adverse events	Active, not recruiting
	Nivolumab plus bevacizumab, surgery			
	Nivolumab plus ipilimumab, surgery			
NCT02853331	Pembrolizumab plus axitinib	III	PFS and OS	Active, not recruiting
NCT03680521	Nivolumab plus sitravatinib	II	Objective response rate	Recruiting
NCT03141177	Nivolumab plus cabozantinib	III	PFS	Active, not recruiting
NCT02014636	MK-3475 plus pazopanib	I, II	PFS and incidence of adverse events	Completed
NCT01984242	Atezolizumab plus bevacizumab	II	PFS	Completed
NCT02420821	Atezolizumab plus bevacizumab	III	PFS and OS	Active, not recruiting
NCT02684006	Avelumab with axitinib	III	PFS and OS (PD-L1+)	Active, not recruiting
NCT02811861	Lenvatinib plus everolimus or Pembrolizumab	III	PFS	Active, not recruiting
NCT02493751	Avelumab plus axitinib	I	Number of participants with DLTs	Active, not recruiting

*The information of this table was obtained from http://clinicaltrials.gov/ and previous studies. IO, immunotherapy; RCC, renal cell carcinoma; PFS, progression-free survival; OS, overall survival; DLT, dose limiting toxicities*.

As mentioned before, tumor escape closely correlates with immunosuppression in overcoming immune response. Whether immune TME and tumors are friends or foes, immunotherapy and targeted therapy may not always get along perfectly, despite the seemingly decent results reported presently. Therefore, how to turn the foe into a friend and match them into an optimal “combo” has become a new concerned topic of further studies. Accordingly, the most potential combination treatment should meet one of the following requirements: (a) ICIs plus immune TME-modifying and anti-angiogenetic agents; (b) immune TME-modifying agents plus anti-angiogenetic agents; and (c) adoptive T cell therapy plus anti-angiogenetic agents (88, 89).

#### Angiopoietin/Tie2 Agonist and Interferon-γ-Related Regulator

We have mentioned that Ang/Tie2 pathway may be involved in the resistance to targeted therapy. Some preclinical trials (77) demonstrated that the benefit of anti-VEGF and anti-Ang/Tie2 was superior to that of monotherapy and could be facilitated by PD-1 blocker. The infiltration of TAMs, DC, and IFN+ CD8+ T cells ascended with the increased frequency of PD-L1. Interestingly, overall survival (OS) improved in over 30% mice. As the combination of anti-PD-1, anti-VEGF, and anti-Ang/Tie2 could reverse immunosuppression and promote immune response, it is worthy of expectation for the treatment of RCC.

#### Stimulator of Interferon Genes Activators

Natural immunity should not be underestimated, such as stimulator of interferon genes (STING), one of the important regulators of natural immunity. Emerging data indicate that STING can mediate anti-immunosuppression, and STING activators can enhance immune response (90). STING activators can promote IκB kinase (IKK)/TRAF-associated NF-κB activator (TANK)-related pathways and stimulate IFN and NF-κB in the immune TME, thus increasing the level of inflammatory cytokines (91). In addition, a clinical trial on STING activators with or without ICIs in advanced solid tumors (MK-1454-001) is underway. We would like to predict that STING-targeted therapies could enhance antitumor immunity. Further studies of STING regulation is required to identify the circumstances where RCC benefits from STING activators and enable more precise drug design.

#### Colony-Stimulating Factor 1 Receptor Inhibitors and Targeted Therapy

In a large multi-transcriptomic analysis of an RCC cohort (92, 93), the results of angiogenesis and macrophage clustering demonstrated that patients with high angiogenesis score clusters and low macrophage infiltration scores could benefit from targeted therapy alone, and patients with high angiogenesis score clusters and high/low macrophage infiltration scores could obtain more benefits from targeted therapy combined with macrophage-targeted immunotherapy, such as colony-stimulating factor 1 receptor (CSF1R) inhibitors, while patients with low angiogenesis score clusters and high macrophage infiltration scores could gain more from CSF1R-directed inhibitors alone. Although only one type of inhibitor (CSF1R inhibitors) was mentioned in their speculation from the results, the conclusion was intriguing because of the promising positive immune-regulated effect of macrophage inhibitors in the immune TME.

#### Chimeric Antigen Receptor T Cells and Targeted Therapy

Adoptive transfer data (94) indicated that the combination of carbonic anhydrase IX (CAIX)–chimeric antigen receptor T cell (CAR-T) therapy and sunitinib defensed against lung metastasis of human RCC in a mouse model and improved the survival, but it increased the frequency of T cell infiltration in the TME. Another clinical study (95) showed that CAIX–CAR-T led to antigen-specific response and liver toxicity in patients with the 2 × 10^8^ T cells as the lowest dose. The result in their 12 patients with mRCC receiving CAR-T cells demonstrated that blockage of the antigenic target could decrease the toxicity and help patients tolerate higher doses. More importantly, the concept of CAR-T therapy may be expanded that targeted therapy synergizes the efficacy of CAR-T, where sunitinib could upregulate intratumoral CAIX and decrease MDSCs (94) in the TME.

### The Adverse Events of Combination Therapy

The main reason for friends becoming foes originates from the adverse events. An overactive immune response often arises from T cell storm and hyperactive inflammation. The modification of immune balance could lead to injury to other organs or tissues, especially liver toxicity (15, 96), unless the doses are decreased in the combined therapy (14). In addition, the attenuation of tumor neovascularization helps T cell infiltration and drug delivery. Thus, anti-angiogenetic agents potentially aggravate adverse events of immunotherapy (14, 97).

## Conclusion

Increasing evidence has demonstrated that it is difficult to determine whether immune TME and targeted therapy are friends or foes in a particular setting. However, preclinical and clinical trials have demonstrated that immunotherapy and anti-angiogenetic drugs can work synergistically. We speculate that this situation results from the following two aspects: (1) immunotherapy attenuates immunosuppression and decreases tumor vascularization; and (2) anti-angiogenetic drugs reduce the percentage of immunosuppressive cells, thus blocking immune escape. The dominant challenge is to find an optimal combination of these two treatments and obtain the optimal dosage of combined therapy for the sake of magnifying the clinical benefits. In addition, how to eliminate resistance and improve the prognosis is also an important issue that needs to be considered seriously in developing a promising therapy.

## Author Contributions

All authors had full access to all the data in the study and take responsibility for the integrity of literature. XC, WC, and XP contributed to reviewing the concept and design. WC and XP contributed to drafting the manuscript. All authors were involved in critical revision of the manuscript for important intellectual content.

## Conflict of Interest

The authors declare that the research was conducted in the absence of any commercial or financial relationships that could be construed as a potential conflict of interest.
